# A Pleiotropic Nuclear Hormone Labelled Hundred Years Ago Vitamin D

**DOI:** 10.3390/nu15010171

**Published:** 2022-12-30

**Authors:** Carsten Carlberg

**Affiliations:** 1 Institute of Animal Reproduction and Food Research, Polish Academy of Sciences, PL-10-748 Olsztyn, Poland; c.carlberg@pan.olsztyn.pl; 2 School of Medicine, Institute of Biomedicine, University of Eastern Finland, FI-70211 Kuopio, Finland

This year we are celebrating 100 years of the naming of vitamin D, but the molecule is, in fact, more than one billion years old [1]. *Nutrition Bulletin* and *Endocrine Connections* are also honoring the centenary of vitamin D’s discovery, but with 21 original publications and review articles written by experts in the field, *Nutrients* represents the largest collection [1,2,3,4,5,6,7,8,9,10,11,12,13,14,15,16,17,18,19,20,21].

At the beginning of the last century, small molecules were found to cure several diseases caused by nutritional deficiencies and were, therefore, termed vitamins. These diseases are xerophthalmia (a clinical spectrum ranging from night blindness to complete blindness), anemias, and neurological disorders, such as beriberi and scurvy (a disability affecting the repair of bone, skin, and connective tissue), which can be healed and prevented using the supplementation of vitamins A, B and C, respectively. Thus, when McCollum and colleagues demonstrated in 1922 that experimentally induced rickets in rats could be cured by a factor isolated from cod liver oil, they followed the nomenclature termed and named it vitamin D [22].

The term “vitamin” implies that these molecules should be regularly supplied as part of our diet or as pills. However, some vitamins can be endogenously produced by our bodies, such as vitamin D_3_ in UV-B-exposed skin [23]. Thus, changes in our lifestyle, such as spending more time indoors, wearing clothes outdoors, and living at latitudes with seasonal variations of UV-B intensity, made vitamin D a vitamin. The insufficient production or supplementation of vitamin D_3_ causes a low vitamin D status, which is determined by the serum concentration of the most stable vitamin D_3_ metabolite, 25-hydroxyvitamin D_3_ (25(OH)D_3_). Severe vitamin D deficiency, defined as 25(OH)D_3_ serum levels below 30 nM (12 ng/mL), can lead to bone malformations, such as rickets in children and osteomalacia in adults [24], and at all ages, lead to a malfunctioning immune system [25]. The latter may increase the risk for severe consequences of infectious diseases, such as COVID-19 (coronavirus disease) [26] or tuberculosis [27], as well as for the onset and progression of autoimmune diseases, such as type 1 diabetes [28] and multiple sclerosis [29].

Vitamin D and vitamin A differ from other vitamins due to the interesting property that a few metabolic steps can convert them into nuclear hormones that bind with high affinity to members of the nuclear receptor superfamily [17]. In the case of vitamin D_3_, this is the metabolite 1,25-dihydroxyvitamin D_3_ (1,25(OH)_2_D_3_) activating the transcription factor VDR (vitamin D receptor) [4]. In fact, 1,25(OH)_2_D_3_ binds to VDR with a K_D_ of 0.1 nM, which is a significantly higher affinity than that of other nuclear receptors for their specific ligands. This suggests that the genomic signaling of 1,25(OH)_2_D_3_ via VDR is the predominant means of vitamin D’s mechanism of action [30]. However, there are also indications for non-genomic vitamin D signaling [19] as well as the biological activity of vitamin D metabolites other than 1,25(OH)_2_D_3_ [16].

In genomic signaling, in all VDR-expressing tissues and cell types, vitamin D controls the transcription of hundreds of target genes [4]. The Genotype-Tissue Expression (GTEx) project provides gene expression data from 54 tissues obtained from 948 post-mortem donors [31]. The selected tissues are representative of more than 400 tissues and cell types that constitute our body. At present, the publicly available dataset (https://gtexportal.org) (accessed on 12 December 2022) is the gold standard for comparing tissue-specific gene expression. The expression of the *VDR* gene is highest in the skin, intestines, and colon and lowest in different regions of the brain (Figure 1). In other tissues, such as the blood and kidneys, the *VDR* gene shows intermediate levels of expression. The shortcut interpretation of this gene expression panel is that vitamin D, via the activation of VDR, primarily impacts the tissue of its synthesis and that its main action is in the gut, while in the brain, it may have no direct function. However, one has to distinguish the role of vitamin D as a controller of calcium transport in the gut [7] from its regulatory function, e.g., in the immune system [3]. Therefore, immune cells may not need as many VDR proteins as intestinal cells.

From an evolutionary perspective, the calcium-absorbing and bone-remodeling function of vitamin D (Figure 2) was obtained less than 400 million years ago, when species left the ocean and needed a stable skeleton [1]. Thus, the best-known role of vitamin D was not why evolution created vitamin D endocrinology. However, regarding this physiological function, VDR and its ligand became dominant regulators [7,20]. In this context, vitamin D learned to control the expression of parathyroid hormone (PTH) and fibroblast growth factor 23 (FGF23) [11]. The peptide hormones are expressed in the parathyroid gland and osteocytes, respectively, and up- and down-regulate the production of 1,25(OH)_2_D_3_ (Figure 2).

Most likely, the first function of VDR was the regulation of energy metabolism [32]. Energy is essential for basically all biological processes, but particularly for the function of innate and adaptive immunity [33]. Vitamin D and its receptor obtained via the control of immunometabolism a modulatory role on immunity [34] (Figure 2).

Taken together, despite the story of its naming, vitamin D is not only a vitamin that prevents bone malformations. In contrast, via 1,25(OH)_2_D_3_, vitamin D is a direct regulator of gene expression, resulting in pleiotropic physiological functions.

## Figures and Tables

**Figure 1:**
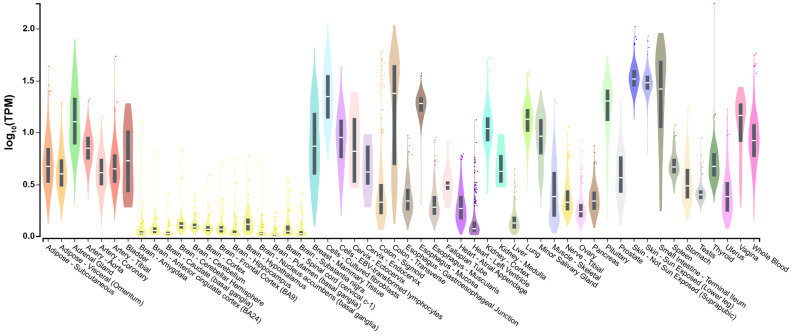
Expression of the *VDR* gene in 54 different human tissues. Normalized RNA sequencing (RNA-seq) data are shown in TPM (transcripts per million), where all isoforms were collapsed into a single gene. Box plots display the median as well as 25th and 75th percentiles. Points indicate outliers that are 1.5 times above or below interquartile range. Data are based on GTEx analysis release V8 (dbGaP Accession phs000424.v8.p2) [31].

**Figure 2:**
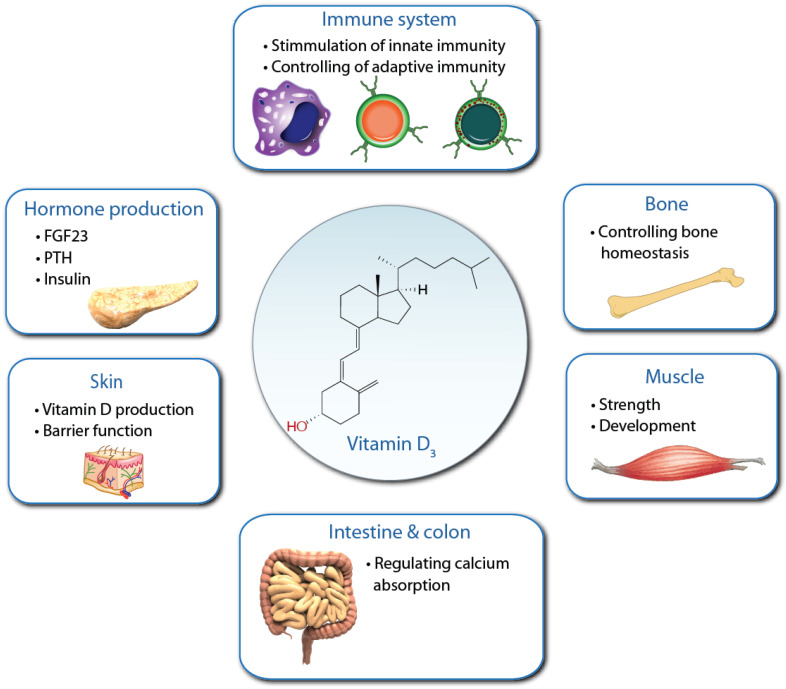
Pleiotropic physiological functions of vitamin D. Vitamin D_3_ (center) can be produced endogenously in UV-B exposed skin. Via its metabolite 1,25(OH)_2_D_3_, vitamin D_3_ activates VDR in various tissues and cell types, where it regulates the indicated major physiological functions.
